# Public Health in the United States.—II

**Published:** 1906-01-20

**Authors:** 


### PUBLIC HEALTH IN THE UNITED STATES.—II.

(*Continued from page* 231.)

### Quarantine.

The measures existing in the United States for preventing the introduction of infectious diseases from other countries into the United States through its principal sea-ports, are provided chiefly by laws enacted by those States which are on the sea coast, and by certain statutes of the general Government which confer authority for inspection on the Marine Hospital Service, a department of the United States Treasury (Act of February 15th, 1893). By this Act the chief official of this department is required to examine the quarantine regulations of State and municipal boards of health, and to co-operate with these authorities in preventing the introduction of disease from abroad, as well as from one State to another. United States officers in foreign ports are also required to publish regulations for securing the best sanitary condition of vessels, cargoes, passengers, and crews departing for the United States. The quarantine and inspection stations—which are conducted either by national, State, or local authorities—are 120 in number, of which 81 are on the Atlantic coast, 22 are on the Gulf of Mexico, and 17 are on the Pacific coast. Several of these ports are of very little importance. By far the most important station as a quarantine port is that of New York, since the arrivals of vessels at that station from foreign ports may be counted annually by thousands, and the number of immigrants by hundreds of thousands. The equipment of this station consists of a boarding station, with the necessary offices for the health officer of the port and his assistants; a sufficient anchorage ground for incoming vessels; two large and powerful steam-tugs for boarding facilities; apparatus and steamer for disinfection of vessels and for bathing immigrants and disinfecting their personal effects; a detention and disinfection station on Hoffman Island—an artificial island of 8 acres, having hospital buildings for the accommodation of 200 patients, a morgue and crematory, and all necessary appliances for hospital treatment of quarantinable diseases. The quarantinable diseases are cholera, yellow fever, small-pox, typhus fever, leprosy, and bubonic plague.

### Food and Drug Inspection.

It was not till a comparatively recent period that public attention was directed to the adulteration of food. The first State which took definite action on the subject was New York, the law having been passed June 15th, 1881. That of Massachusetts followed in 1882, and several other States have enacted similar laws in the succeeding years. In New York and Massachusetts the execution of this law was entrusted to the State boards of health, while in other States, notably in Ohio and New Jersey, it has been placed in the hands of a special food commission, clothed with similar powers and authority. Several States also have dairy commissioners appointed to take charge of the inspection of milk and other dairy products. In some of the States where an adequate appropriation has been made for the execution of the law, it has been enforced, and the results have been all that could be expected. Thousands of samples have been collected and examined, and many offenders have been brought to trial and convicted, while fraudulent and dishonest preparations have been driven across the border into States which have made no provision against the evil.

In addition to the inspection provided by the States, the large cities also usually provide for inspection of milk and other articles of food offered for sale in their own limits. There are also in some of the cities special inspectors of animals, provisions, and markets, acting either under the direction of the health department or of some other bureau. The milk supply of all the large cities of the Union is subject to daily inspection by competent officials, for the purpose of preventing adulteration, and measures are being adopted in some of the large cities to secure an improved condition of the producing dairies, both as to the health of the animals and the conditions under which they are kept.

An Act of Congress, dated August 30th, 1890, ch. 839, provides for an inspection of meats for exportation, and prohibits the importation of certain adulterated articles of food. Its principal provisions are the following:—The inspection of salted pork and bacon intended for export, and to determine whether it is wholesome, sound, and fit for human food. Inspection to be made where the meat is packed. Adulterated food and drugs and liquors injurious to health prohibited, and suspension of imports may be proclaimed by the President. Importation of diseased cattle prohibited; quarantine and slaughter of infected animals provided for. Live animals for export to be inspected, and the diseased not allowed to go out of the country. A later Act of Congress, passed March 3rd, 1891, also provides for the inspection of cattle intended for export, and of those whose meat is to be exported; it also provides for inspection, before slaughter, of animals intended for inter-State commerce. Post-mortems are also provided for. Penalties are also imposed for forging or counterfeiting marks and tags. The transport of unsound carcases is forbidden. The provisions of this law are not to apply to animals killed on the farm.

### Public Water Supplies.

In no department of public hygiene has more rapid progress been made in the United States than in the introduction of public water-supplies in cities and towns. In the eighteenth century very few municipalities had taken any action towards introducing supplies of pure water for the use of the inhabitants, private wells, springs, and cisterns for collecting the rainfall being the principal sources of supply. The whole number of towns supplied before 1800 was 16. In 1850 only 83 water-supplies had been introduced. In 1890 the total number of supplies had increased to 2,074. In 1896 the number had increased to 3,196, and since in many instances a single plant furnished water to several municipalities, the total number of cities and towns thus furnished, either partially or wholly with public water-supplies, was 3,942. During the last quarter of a century several very extensive schemes for supplying water to large populations have been inaugurated. Among them may be named the extension of the water system of New York City by the enlargement of its tributary watershed and the construction of the Croton dam, with the Croton lake and the Jerome Park reservoirs.

Great attention has also been given during the past 25 years to the subject of water purification, and measures have been undertaken, and in some instances completed, in several cities for the purification of their water-supplies by means of filtration. Among these, special mention may be made of the city of Lawrence, Mass., which had for about 20 years drawn its supply directly from a river which was polluted by the sewage of several hundred thousand inhabitants living above the intake of the waterworks. Typhoid fever had increased in the city to alarming proportions. But after the introduction of the filtration system the death-rate from this disease was reduced to less than one-tenth of its former size, and has continued to diminish from the year after such introduction of the new filtering plant. Similar measures are being taken in other American cities.

*(To be continued.)*

